# Use of Titanium Elastic Nails in the Adult Diaphyseal Humerus Fractures

**DOI:** 10.5704/MOJ.1707.019

**Published:** 2017-07

**Authors:** AS Upadhyay, NA Lil

**Affiliations:** Department of Orthopaedics, SMT NHL Medical College and Vadilal Sarabhai General Hospital, Gujarat, India

**Keywords:** fracture shaft humerus, adult, ante grade, intramedullary, titanium elastic nailing

## Abstract

**Introduction:** Humerus shaft fractures in adults can be managed conservatively with functional braces or operatively with plate osteosynthesis or with intramedullary nailing. Conventionally humerus intramedullary nailing is done either using humerus interlocking nails or Enders nails. In this study we analyse the outcome of humerus shaft fractures (upper and middle third) managed with closed ante-grade intramedullary titanium elastic nailing in terms of union rates, union time, functional results and complications.

**Materials and Methods:** A retrospective analysis of 25 patients treated with ante-grade intramedullary titanium elastic nailing was done during the period of August 2014 to June 2016 at our tertiary care centre. Skeletally mature patients with closed and acute fractures of the upper and middle shaft humerus of both sexes were included in the study.

**Results:** Twenty-five (100%) fractures united with an average consolidation time of 14.98 weeks (10-32 weeks), and no fractures ended in non-union. Nail impingement was seen in two (8%), shoulder stiffness in one (4%). Functional results were excellent in 22 (88%), moderate in two (8%) and poor in one (4%). There were 17 males and eight females with an average age of 39.08 years (range: 18-65 years). The average duration of hospital stay was 3.84±2.30 days.

**Conclusion:** Closed ante-grade intramedullary titanium elastic nailing offers a safe and reliable method of fixing fractures of upper humeral shaft in adults. This method provides early fracture union, high union rates and less complications, in addition to early rehabilitation and reduces the hospital stay.

## Introduction

Fractures of the shaft of humerus are commonly encountered by orthopaedic surgeons, representing 1-2% of all fractures^1–3^. This fracture can be treated by either conservatively using functional braces/plaster support or surgically. The two modalities of internal fixation in fracture shaft of humerus are plate osteosynthesis and intramedullary nailing. Nails are subjected to smaller bending loads and are less likely to fail due to fatigue. They act as load sharing and stress shielding devices^1,2^. Cortical osteopenia that occurs right adjacent to the ends of plates is rarely seen with intramedullary nails; thus, refracture after implant removal is seen less often^1^.

This treatment method has been the subject of controversy since its inception because of concern of damage to medullary circulation, possibilities of fat embolism and generally a lack of understanding of the biomechanical principles of intramedullary nail fixation^4,5^.

Intramedullary nails can be interlocking nails, Enders or titanium elastic nails. Titanium elastic nail are used extensively in paediatric long bone fractures but their use in adult fractures is limited to management of clavicle fractures and forearm fractures.

So, we undertook this study to identify the utility, advantages, shortcomings, difficulties, and to prepare guidelines for the treatment of adult fracture upper and middle shaft humerus. The study further aimed to evaluate the functional outcome in terms of range of motion and subjective complaints following the use of flexible intramedullary titanium nail in proximal and middle AO type 12. A1, A2, A3 shaft humerus fracture in adults, the intent being also to study the time to union and rate of union of the fractures along with post-operative complications following the ante-grade titanium nailing.

## Materials and Methods

A retrospective analysis of data, spanning over a period of two years (mean 20 months) from August 2014 to June 2016 was carried out. The data were collected from the Department of Orthopaedics, at our tertiary care hospital. Twenty-five adult patients with traumatic fractures of humeral shaft (AO Type 12. A1, A2, A3) treated with closed ante-grade intramedullary titanium elastic nailing were included in the study.

Adults more than 18 years of age (skeletally matured), closed acute traumatic diaphyseal fractures of humerus involving the upper and middle zones up to 3 cm beyond deltoid tuberosity, canal diameter more 6.5 mm. (digital radiographic computer software AGFA-NX 2.0), fractures morphology – two-part transverse or oblique fractures (spiral and short), patients with head injury not associated with any paraparesis or paraplegia, chest or abdominal injury with shaft humerus fractures were included in the study.

All open fractures, unstable fracture patterns (comminuted, unstable large butterfly fragment), skeletally immature bones, associated radial nerve palsy, pathological fractures, peri-prosthetic fractures, pre-existing shoulder or elbow pathology were excluded from the study.

Preoperative true-size digital radiographs of humerus were taken in anteroposterior and lateral views with shoulder and elbow joints included. Estimation of the narrowest part of the humerus intramedullary canal and approximate length were measured using digital radiograph-based computer software (AGFA-NX 2.0). Primary stabilisation of the fractures in all patients was done by U-slab. Almost all patients were operated within 24 to 48 hours except six patients with associated head/chest injury, and comorbid medical condition were first primarily stabilised and operated thereafter as early as possible.

**Fig. 1:**
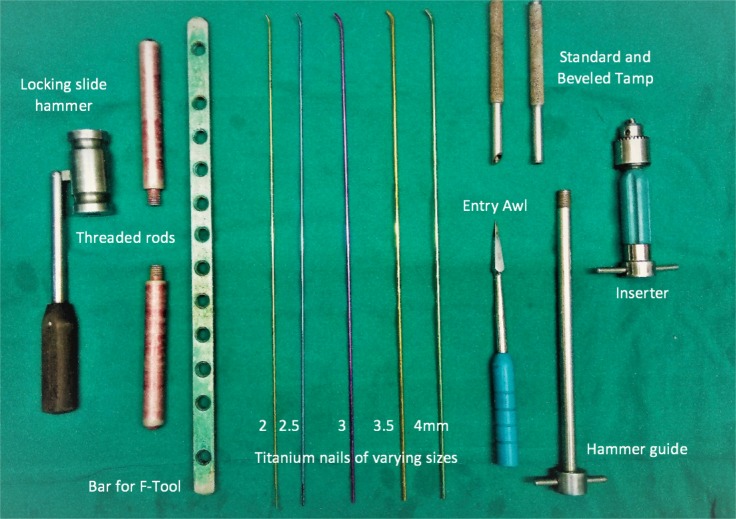
Implants and instruments. Titanium elastic nails of different sizes (4mm-2mm), with entry awl, T-handle nail bending rod. Note: the smooth contoured tip of the nail (the sliding surface) which allows the smooth sliding of the nail in the canal without breaking it.

Titanium elastic nails (Ti-6A1-7Nb titanium alloy) of varying sizes. (2 mm to 4 mm) (Nebula Ortho Care, Ahmedabad, India.) were used. (Fig. 1)

**Fig. 2:**
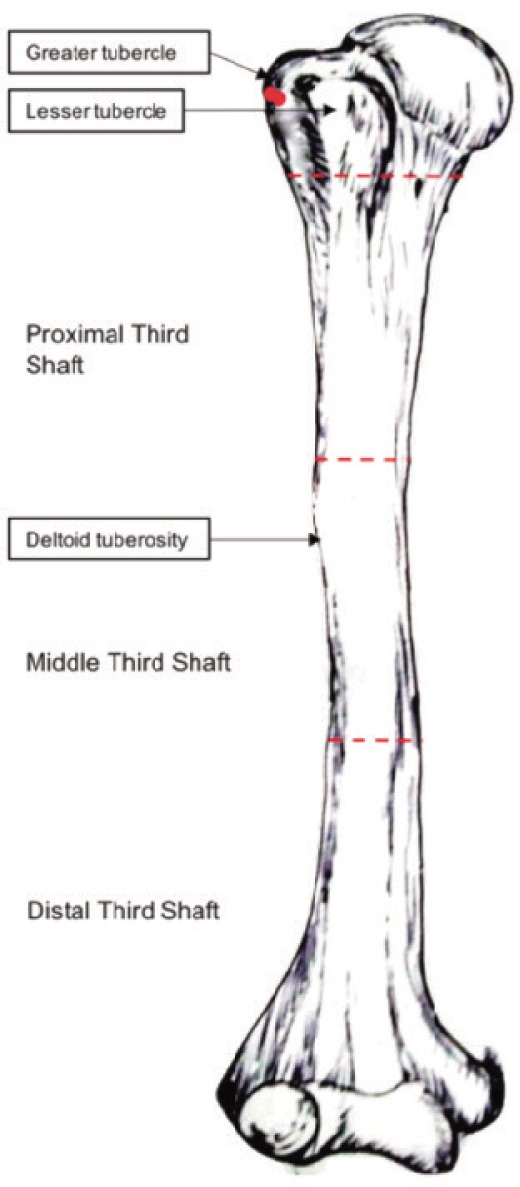
Topographical classification of humerus. The red dots indicate the nail entry point.

Patients were placed in supine position under anaesthesia on radiolucent table with a pack below the scapula. All the cases were treated by closed intramedullary titanium elastic nailing with an ante-grade entry point. “C” contouring of the nail (nail contoured into a bow with nail tip pointing to the concave side of the bowed nail) was done before insertion so to achieve three-point fixation. (one at the entry site, one intramedullary at the fracture site and one at the tip of the nail distally). Entry point was at the greater tuberosity just lateral to the insertion of supraspinatus tendon, through a lateral incision approximately 2 cm long, after splitting the deltoid in line of its fibres (Fig. 2). Two titanium elastic nails of appropriate size so as to fill the canal were inserted with two separate entry points through the same incision.The nails were negotiated across the fracture site after closed reduction under fluoroscopic guidance. Distal fanning of the nail tips were done medially and laterally in the coronal plane fixing in the medial and lateral pillar. Proximal ends of the nails were cut flush to the bone and buried in it.

**Fig. 3:**
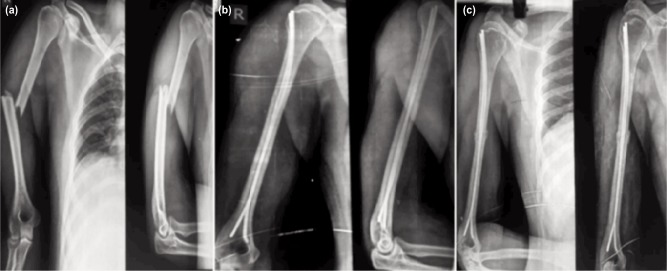
Pre op, post op and follow up radiographic images (anterior-posterior and lateral) through various phases of union. Note the contour of the nail (C- bending) and distal fanning in both the planes, (a) preoperative radiograph, (b) immediate post-operative radiograph and (c) final follow-up radiograph at the end of 2 years union at the fracture site.

All patients were given humerus U-slab in the post-operative period and elbow mobilisation with slab was started from the second post-operative day. Stitches were removed on the 10th post-operative day and functional humerus bracing applied. Patients were given physiotherapy in form of biceps and triceps strengthening exercises, static and dynamic along with shoulder pendular exercise with the humerus braces. Patients were regularly followed-up at four weeks, eight weeks, ninth months and at one year and two years (mean follow-up of patients was 20 months). Radiographs were taken during follow-ups for clinical and radiological assessment of fracture healing. (Fig. 3).

The clinical criteria for fracture healing was absence of pain and tenderness at fracture site with free shoulder and elbow activity. Radiological criteria were presence of bridging callus and disappearance of the fracture line on serial follow-ups. The functional results were recorded according to the CONSTANT shoulder evaluation form^6^.

## Results

The following observations were made during follow-up of all the 25 cases for a minimum period of 18 months:

Age range of our patients was from 18 years to 65 years with an average of 39.08 years. The majority of patients 17 (68%) were males and only 8 (32%) were females. Road traffic accident was the commonest mode of injury accounting for 18 (72%) patients, the remaining six (24%) patients presented with the history of fall and one (4%) gave a history of assault. In our study, 19 (76%) patients had fracture at middle third of shaft of humerus and six (24%) patients had fracture at proximal third of shaft of humerus. Sixteen (74%) patients had transverse fracture, seven (28%) had oblique fracture, and two (8%) had spiral fracture. Nineteen patients were operated within two days of trauma, six patients had a delay in definitive fixation and two patients had head injury and were operated on the 4th and 7th day. One patient had a chest injury and was operated on the4th day. Three patients with uncontrolled medical comorbidities were operated after their condition stabilised (after 5-7 days). The average time interval between admission and surgery was 2.28±1.89 days. The duration of surgery was calculated from the start of incision to skin closure, and was 37.2 ±8.78 minutes. The average duration of hospital stay was 3.84 ±2.30 days.

**Fig. 4:**
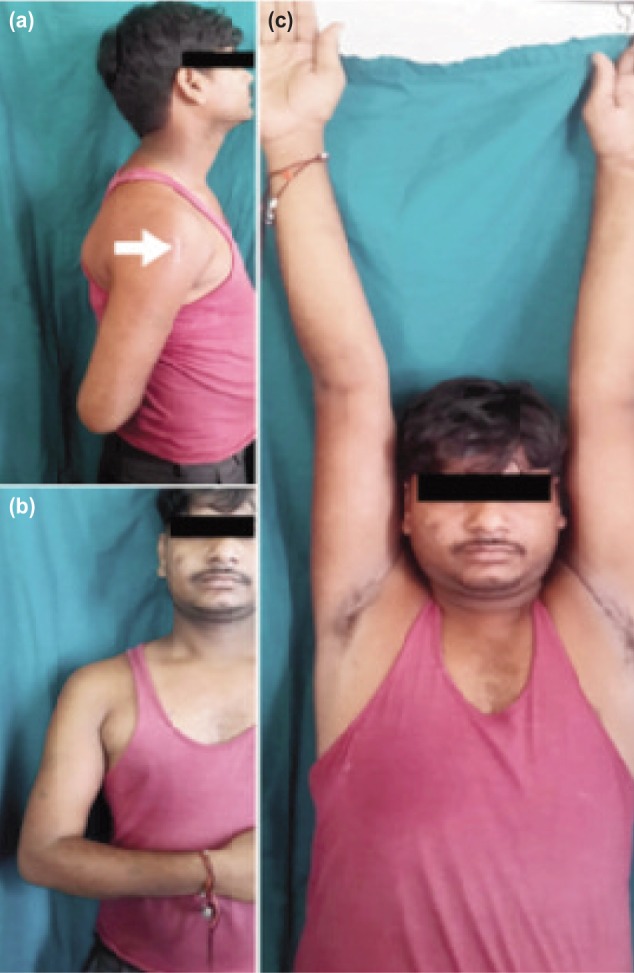
Clinical photographs at end of follow-up demonstrating complete range of motion; (a,b) internal rotation and (c) overhead abduction. Note the small incision white arrow on the right shoulder (a).

The union rate was 100% in the present study at the final follow-up. The period of fracture union ranged from 10 weeks to 32 weeks, with an average period of 14.98±4.08 weeks. There was a single case of delayed union in the present study which eventually healed at 32 weeks without any intervention. There were no cases of non-union in this study. No bone grafting was done in any patient either primarily or secondarily. As regards to functional assessment of patients, shoulder function was excellent in 22 (88%) patients, good and fair in two (8%) patients and poor in one (4%) patient. Elbow function was excellent in all patients (Fig. 4) (Table I).

**Table I:** The final outcome in terms of functional score (constant score), subjective symptoms and radiological assessment. 88% had an excellent functional score with good radiological union and symptoms at the end of follow-up

**Functional score (constant score)**	**Loss of range of motion**	**Radiological union**	**Subjective symptoms**	**Number of cases**
Excellent	<10%	Good	No	22
Good and fair	10-30%	Good	Mild	2
Poor	>30%	Good	Moderate	1

Two (10%) patients had nail impingement at the proximal end as it was not buried completely into the bone. There was restriction of shoulder movement (terminal 20 degrees of abduction), and were considered to have moderate functional outcome. One (4%) patient ended up with shoulder stiffness mainly affecting abduction (0-60 degree) and internal rotation (up to lumbosacral junction) at end of 14 months. This was due to reflex sympathetic dystrophy which was clinically evident by muscle wasting, shiny skin, loss of hair and increase in local perspiration. There were no cases of iatrogenic radial nerve palsy or iatrogenic comminution during nail insertion in our study.

## Discussion

The goal of fracture management is to restore anatomy and physiological functions. Treatment of humerus fractures is dictated by certain anatomical factors, as outlined by Mast *et al*^8^. The humerus is not a weight-bearing bone and compressive forces are not as significant a factor in healing as in other long bones, such as the femur and tibia. Closed treatment of humeral shaft fractures represents an effective method of fracture management and has been the subject of critical evaluation throughout the literature^9,10^. Very frequently, they are associated with multiple injuries, leading to complications like shortening, mal-union, infection, delayed union and non-union. Operative treatment may be considered to avoid complications such as mal-union, delayed union, rotational deformity, shoulder and elbow stiffness, limb length discrepancy and psychological problems^11^. The operative intervention includes a number of surgical procedures like open reduction and rigid internal fixation (plate osteosynthesis) and closed reduction and internal fixation with locked intramedullary nail (humerus interlocking nail, Seidels nail, etc.) and elastic flexible intramedullary nail (the titanium elastic nail, the Enders nail) with each having its own drawbacks. Fixation with plate leads to extensive soft tissue dissection, incidences of iatrogenic radial nerve palsy, higher incidences of wound morbidity and infection. Previous studies by Heim *et al* showed a 5% incidence of infection with the use of plate osteosynthesis^12^.

Kumar in his study on patients treated with plate osteosynthesis reported 13.3% rate of both infection and transient radial nerve injury^13^. Studies on adult diaphyseal fractures of humerus with intramedullary nailing have all reported results involving interlocking nail, Russell nail, Enders nail, etc. A few studies have been reported on the use of titanium elastic nail in the management of humeral fractures of adults. Hems and Bhullar suggested that ante-grade interlocking nail affects fracture healing by distracting the fracture and soft tissues leading to a higher incidences of non-union^14^. Rosenbaum and Uhl reported that the incidences of non-union with the use of flexible nail was as high as 8-10%^15^. There was no incidence of non-union in our study, similar to the study by Sleman, in which there was no incidence of non-union in humeral diaphyseal fractures with titanium elastic nail^16^. From the above stated studies it is evident that fracture distraction is one of the important causes of delayed union and non-union of fracture shaft of humerus specially with the use of nails. Modi *et al* compared the outcome of titanium elastic nail and plate in the adult shaft humerus fractures and concluded that results of titanium elastic nail in union rates and union time were good and comparable to plates with lesser rate of complications, but their usefulness is limited to middle third fractures of humerus. The results of titanium elastic nail depend greatly on jamming of canal, control of rotation, and proximity of fracture to entry point^17^.

Ante-grade nailing in the humerus has always raised the issue of shoulder impingement or rotator cuff damage^18–20^. There has also been a report about patients with diaphyseal humeral fractures who were treated with retrograde nailing without delay but developed stiffness and discomfort of the shoulder joint^21^. This report of shoulder impairment occurring even when there has been no direct surgical intervention to the joint suggest that shoulder pathology may occur after a humeral shaft fracture regardless of the treatment method and ante-grade intramedullary nailing may not always be the precipitating cause of shoulder discomfort and functional impairment. In our study, there were only two patients who had complained of shoulder pain and stiffness; otherwise 92% of the patients achieved painless full range of shoulder movement. We emphasize the essentiality of proper entry of nail in order to avoid damage to the rotator cuff and burying of the nail in the proximal humerus to minimise impingement.

Most surgeons agree that intramedullary nailing is the best internal fixation for femoral and tibia shaft fractures, but there is no agreement on the ideal procedure for fractures of the humeral shaft. The use of intramedullary nailing has increased the union rates, reduced mal-union and offers functional outcomes that are comparable to all nail designs. Brumbeck *et al* reported 2.2% incidence of iatrogenic comminution at the fracture site with the use of Enders nail, and Jinn reported a 5.5% incidence of iatrogenic fractures with the use of Seidel interlocking nail^19,22^. Our study showed no incidence of iatrogenic fractures, as titanium has modular of elasticity nearer to the human bone, whereas Enders nail and interlocking nail which are made of stainless steel are stiffer. As a result titanium nails are easier to negotiate through the bone. As they bend while passing through the bone tension is increased within the nail which improves the three-point fixation.

The key distress with respect to titanium elastic nail is its inability to provide suitable rotational stability. The rotational instability can be overcome to an extent with the pre-insertion “C” contouring of nail providing an efficient three- point fixation, distal fanning of nail tips and different entry points for nail insertion. The other apprehension is the distraction at the fracture site in the follow-up, as the humerus is a “hanging” bone and subject to gravitational forces. This can be managed simply by post operatively giving humerus a U-slab or functional brace and early allowing of elbow physiotherapy with brace providing a hydraulic effect through the biceps and triceps contractions. With the above known setbacks and identified methods to handle them, the use of titanium elastic nails yielded excellent functional outcome within 10-16 weeks with very limited complications. The non-ferromagnetic property of titanium nail further enhances its advantage as it will never interfere with any future MRI (if required). It also reduces the need for a second surgery for implant removal.

We acknowledge that our study has some limitation as the sample size is small and long term follow-up is awaited. We do not recommend the use of titanium elastic nails in distal humeral shaft fractures, communited fractures or in unstable fracture pattern.

## Conclusion

Intramedullary nailing of the humerus using titanium elastic nail for short oblique and transverse fracture of adult upper and middle ( AO type 12 A1 A2 A3.) shaft humerus is an effective surgical modality, with advantages of minimal incisional scar, minimal soft tissue damage, with early union time and excellent union rates, and without affecting shoulder and elbow function.

## Conflict of Interest

The authors declare no conflicts of interest in the preparation of the manuscript.
